# Straight from the Horse’s “Mouth”: Genomic Epidemiology of an Icelandic Equine Epidemic

**DOI:** 10.1128/mBio.01613-17

**Published:** 2017-10-10

**Authors:** Mark R. Davies

**Affiliations:** Department of Microbiology and Immunology, at the Peter Doherty Institute for Infection and Immunity, The University of Melbourne, Melbourne, Victoria, Australia

**Keywords:** *Streptococcus equi* subsp. *zooepidemicus*, equine, genome analysis, genomic epidemiology, outbreak, respiratory pathogens, zoonotic infections

## Abstract

Despite tight biosecurity measures, an outbreak of respiratory disease rapidly spread across the Icelandic equine population in 2010. Horse transportation was brought to a halt in order to contain the spread of the infectious agent. In a recent article, Björnsdóttir and colleagues (S. Björnsdóttir et al., mBio 8:e00826-17, 2017, https://doi.org/10.1128/mBio.00826-17) employ the power and resolution of “genomic epidemiology,” the combination of whole genomic sequencing and epidemiological approaches, to examine the source and spread of the outbreak. Intriguingly, the outbreak was not viral in origin, but linked to a bacterial “commensal” *Streptococcus equi* subsp. *zooepidemicus* infection. A national sampling strategy coupled with population genomics revealed that the outbreak was most likely driven by a *S. equi* subsp. *zooepidemicus* sequence type 209 (ST209) infection that spread nationally from a single source. This retrospective study demonstrates the power of genomics applied on a national scale to unravel the cause of a significant biosecurity threat.

## COMMENTARY

Reports of respiratory outbreaks in livestock elicit national and global concerns of potential pandemics, such as those associated with influenza infections (reviewed in reference 1; also see reference 2). Such scenarios can have devastating, industry-wide consequences. Some geographical regions of the world maintain strict biosecurity practices to prevent the importation and spread of infectious agents in key primary industries, such as the practices of the Icelandic equine industry, where the importation of horses has been banned since 1882. However, in early April 2010, an epidemic of respiratory disease spread across the 77,000-member Icelandic equine population. Such outbreaks can have substantial economic effects for a country that relies on livestock transfers and export. Immediate biosecurity measures were implemented by health authorities, who imposed a ban on the export of horses. Yet by the time the alarm was raised, disease symptoms, recorded through electronic questionnaires sent to hundreds of equine breeding farms and professional training centers, were already widespread.

Such rapid spread of a respiratory infection within a large geographical context is typical of a viral agent; however, PCR-driven analyses of clinical samples did not support carriage of known equine viral respiratory pathogens from infected horses. In a recent article by Björnsdóttir et al., the scientific team analyzed nasal swabs from 100 horses at 31 different geographical sites to identify the bacterium *Streptococcus equi* subsp. *zooepidemicus* as a common link (3). Primarily considered a commensal organism of equine upper airways, *S. equi* subsp. *zooepidemicus* is an opportunistic pathogen in a wide variety of mammalian hosts, including horses, and is capable of causing zoonotic infection in humans (4). Notably, no *S. equi* subsp. *equi*, the classic respiratory equine pathogen and the causative agent of strangles, was isolated from clinical samples, suggesting an underlying role for *S. equi* subsp. *zooepidemicus* in the epidemic. This is not the first time *S. zooepidemicus* has been linked to a veterinary outbreak. Equine outbreaks of *S. equi* subsp. *zooepidemicus* have previously been documented within New Caledonia (5) and Scandinavia (6) and also within other hosts, such as dogs (7, 8) and pigs (9), but not at the national scale as documented by Björnsdóttir and colleagues in Iceland (3).

With advancements in the speed, throughput, and cost of genome sequencing, population analyses of infectious disease outbreaks in the public health sector (primarily foodborne) are routinely driven by whole-genome sequencing (10). Such methodologies offer unprecedented resolution in determining outbreak sources and transmission pathways when quality sampling and epidemiological data are integrated in the study design (11). The dissection of the veterinary outbreak by Björnsdóttir and colleagues is a prime example of informed study design examining the genetic relationship of isolates from clinical outbreak cases, as well as historical, nonequine, and contemporary *S. equi* subsp. *zooepidemicus* isolates for context. Sampling was spread across 31 geographical sites represented in a national survey. A total of 305 *S. equi* subsp. *zooepidemicus* isolates were whole-genome sequenced, of which 257 were isolated during the 2010 equine outbreak period. The well-designed study, including the large sample number and several control sequences, coupled with high-resolution population genomics, allowed the team to draw their epidemiological conclusions.

Key findings from this study identified that *S. zooepidemicus* sequence type 209 (ST209) was present in half of the disease isolates from the majority of infected farms. Three other *S. zooepidemicus* sequence types were also variably present within some clinical samples, albeit, less frequently and more geographically constrained; the authors suggest that these may represent resident endemic strains. Interestingly, the authors found examples of multiple *S. zooepidemicus* sequence types residing within 15 of the infected horses. Such multiclonality has been observed from horses who carried *S. equi* subsp. *zooepidemicus* asymptomatically, defined as the “commensal” state (12), which raises some interesting questions around the topic of multiclonality within clinical cases. Such coinfection by different strains is understudied in clinical microbiology and highlights the need for interrogation of multiple isolates per sample to address the possible role of multiclonality during infection. This is particularly relevant to the Icelandic outbreak, where it appears both endemic (non-ST209) and outbreak pandemic (ST209) strains are circulating within the population concurrently, leading to challenges in disease causation inferences. Furthermore, the transient nature of bacterial colonization suggests that the disease causative agent may not be captured within a nasal swab at the time a diagnostic test is undertaken. One possible way of addressing multiclonality within clinical samples is to implement metagenomics-based approaches in diagnostic microbiology, where samples are sequenced straight from a clinical sample, such as those used in the 2011 Shiga-toxigenic *Escherichia coli* outbreak in Germany (13). Such culture-independent methodologies have the added benefit of amplifying sequences from novel or potential nonculturable microorganisms as well as defining microbial communities to a strain resolution as undertaken in human microbiome studies (14).

Various genomic signatures investigated by the authors support *S. equi* subsp. *zooepidemicus* ST209 as the causative agent of the outbreak. One hallmark of a classic bacterial outbreak strain is low levels of genome-wide sequence variation between isolates from infected individuals. Indeed, the authors identified that the ST209 lineage exhibited a significantly lower divergence in sequence variation than the other “endemic” sequence types in circulation. However, this generalization can potentially be an oversimplification of reality, as demonstrated in human streptococcal outbreaks, such as the ongoing *Streptococcus pyogenes* scarlet fever outbreak in Hong Kong, where the outbreak has been associated with multiple genetically distinct lineages of the same sequence type despite a rapid clinical outbreak of disease (15). Additionally, ST209 was shown to be actively transmitted during the outbreak to uninfected horses within an infected farm. Despite the technical and time constraints associated with such experimentation during an outbreak situation, these results highlight the high transmissibility of *S. equi* subsp. *zooepidemicus* at the time of the outbreak.

One objective from a public health perspective is to identify the possible point source of the outbreak. By applying Bayesian modeling estimates to the sequenced *S. equi* subsp. *zooepidemicus* population, the authors estimated the introduction of the ST209 progenitor to be ca. 2008, suggesting a single introduction of the “new” genotype within a proposed immunologically naive population. Furthermore, through integrating population structure with GPS (Global Positioning System) coordinates of the isolates from infected animals, the authors were able to propose an outbreak transmission pathway. These data led the authors to suggest an equine water treadmill at a single location as a potential point source, yet no isolates were identified from the environmental source in question in order to confirm the link.

Another alarming finding from the study is the ST209 outbreak genotype within a human zoonotic infection during the outbreak period. Notably, two other *S. equi* subsp. *zooepidemicus* samples isolated from humans during the outbreak were non-ST209 strains. This in itself is not unheralded, given that *S. zooepidemicus* is a known opportunistic pathogen of humans, but the presence of the outbreak clone in multiple hosts introduces the possibility that the source of infection may have been transmitted from another host, such as human. In further support of this possibility, one additional human ST209 isolate from Finland appeared to have diverged from a common ancestor with the Icelandic outbreak lineage; however, directionality cannot be inferred with the sample strategy employed. The outbreak itself did not lead to a reported surge in human cases, but the role of a human (or other) reservoir is possible with this outbreak. Such interhost transmission pathways have been proposed in other animal infections, such as *Staphylococcus aureus* (16, 17) and *Salmonella enterica* serovar Typhimurium (18). Irrespective of this point, what this study highlights is that no matter how strict the biosecurity regulations are regarding restricting the importation of “foreign” animals, carriage by an intermediate host is near impossible to safeguard against. This scenario does reiterate the need for high sanitary standards within animal practices. Importantly, this study also highlights the importance of banking isolates from both symptomatic and healthy animals, as without context, the ability to draw sound conclusions from constantly evolving bacterial populations is restricted.

Whether *S. zooepidemicus* ST209 is a globally disseminated clone and what genetic factors within the genome of ST209 isolates may account for the severity of disease remain unknown. Bacterial factors carried by mobile genetic elements may drive a common fitness advantage across different bacterial genetic backbones, irrespective of sequence type, as has been reported in recent *Streptococcus pyogenes* outbreaks (15, 19), and as such, comparative analyses may yield important insight into intersequence-type bacterial outbreaks. Such targeted analyses can be applied to advance diagnostics and surveillance of key clones, as has been applied to *Mycobacterium chimaera* (20). The authors of this research speculate that the infected equine population lacked protective immunity to the outbreak strain, and this remains an interesting hypothesis. What drives an opportunistic commensal pathogen to cause disease is likely to be an intricate balance between host immune status, the genetics of the microorganism itself, and the environment (for example, microflora or coinfection). A community-wide shift in any of these elements could be enough to kick a commensal into gear. Elucidating these factors is key to understanding the drivers of outbreaks that in turn can be used in pathogen surveillance and diagnostics.

Collectively, the work by Björnsdóttir and colleagues highlights the power and utility of genomic epidemiology to investigate microbial forensics within a nationwide veterinary outbreak (3). Challenges remain in linking disease outbreaks with commensal-associated pathogens, yet this study highlights the value of a national reporting system and the benefit of an informed sampling strategy in identifying patterns of pathogen emergence and dissemination. Moving forward, the integration of public health genomics within both veterinary and human communities is key to unravelling the drivers of infectious disease outbreaks, as is being implemented in foodborne infectious disease outbreaks. Such management of infectious diseases at both the national and international levels relies on coordination and knowledge sharing across animal and human health agencies under the auspices of a “one-health” approach to address the intertwined relationship between veterinary, human, and zoonotic disease.
